# Scalar nanostructure of the *Candida albicans* cell wall; a molecular, cellular and ultrastructural analysis and interpretation

**DOI:** 10.1016/j.tcsw.2020.100047

**Published:** 2020-11-08

**Authors:** Megan D. Lenardon, Prashant Sood, Helge C. Dorfmueller, Alistair J.P. Brown, Neil A.R. Gow

**Affiliations:** a Aberdeen Fungal Group, Institute of Medical Sciences, University of Aberdeen, Foresterhill, Aberdeen AB25 2ZD, Scotland, UK; b Molecular Microbiology, School of Life Sciences, University of Dundee, Dundee DD1 5EH, Scotland, UK

**Keywords:** Fungal cell wall ultrastructure, Chitin, β-glucan, *N*-mannan, Cell wall proteins, AFM, atomic force microscopy, BSA, bovine serum albumin, ChBD, chitin binding domain, CWPs, cell wall proteins, EndoH, endoglycosidase H, Fc-dectin-1, soluble chimeric form of dectin-1, GPI, glycosylphosphatidylinositol, HPF/FS, high pressure freezing/freeze substitution, HuCκ, human kappa light chain, NMR, nuclear magnetic resonance, OD_600_, optical density at 600 nm, PAMPs, pathogen associated molecular patterns, PBS, phosphate buffered saline, PRRs, pattern recognition receptors, rpm, revolutions per minute, scAb, single chain antibody, SEM, scanning electron microscopy, TEM, transmission electron microscopy, WGA, wheat germ agglutinin, 2°, secondary, 2D, two dimensions, 3°, tertiary, 3D, three dimensions, 6xHis, hexahistidine tag

## Abstract

Despite the importance of fungal cell walls as the principle determinant of fungal morphology and the defining element determining fungal interactions with other cells, few scalar models have been developed that reconcile chemical and microscopic attributes of its structure. The cell wall of the fungal pathogen *Candida albicans* is comprised of an amorphous inner skeletal layer of β(1,3)- and β(1,6)-glucan and chitin and an outer fibrillar layer thought to be dominated by highly mannosylated cell wall proteins. The architecture of these two layers can be resolved at the electron microscopy level, but the visualised structure of the wall has not yet been defined precisely in chemical terms. We have therefore examined the precise structure, location and molecular sizes of the cell wall components using transmission electron microscopy and tomography and tested predictions of the cell wall models using mutants and agents that perturb the normal cell wall structure. We demonstrate that the fibrils are comprised of a frond of *N*-linked outer chain mannans linked to a basal layer of GPI-proteins concentrated in the mid-wall region and that the non-elastic chitin microfibrils are cantilevered with sufficient lengths of non-fibrillar chitin and/or β-glucan to enable the chitin-glucan cage to flex, e.g. during morphogenesis and osmotic swelling. We present the first three-dimensional nano-scalar model of the *C. albicans* cell wall which can be used to test hypotheses relating to the structure–function relationships that underpin the pathobiology of this fungal pathogen.

## Highlights

• In wild type *C. albicans* yeast cells grown in standard lab conditions:
• Chitin microfibrils are interspersed throughout the inner layer of the cell wall.
• Cell wall proteins are embedded throughout the inner layer of the cell wall.
• The outer fibrillar layer represents *N*-mannan outer chains.
• The length of fibrils correlates with the length of the α(1,6)-*N*-mannan backbone.
• Side chains extend from the α(1,6)-backbone at fixed angles every 10 mannose residues.

## 1 Introduction

The cell surfaces of microbial pathogens define their shape and dictate how they interact with their host. The most common serious fungal pathogen of humans is *Candida albicans*, which accounts for around 100 million mucosal infections and 400,000 systemic infections per year (Brown et al., 2012, Denning et al., 2018).

The *C. albicans* cell wall is an essential and dynamic structure which is modulated to generate elliptical and tubular shapes that define a wide range of morphologies in which this fungus can grow (da Silva Dantas et al., 2016, Gow and Yadav, 2017). The cell wall also provides protection from physical and chemical insults and is involved in interactions with the host immune system via the glycoconjugates that make up the wall. Its components define pathogen associated molecular patterns (PAMPs) which are recognised by pattern recognition receptors (PRRs) that are expressed on the surface of innate immune cells (Erwig and Gow, 2016). Understanding precisely how the PAMPs are arranged in the wall, and how the architectural arrangements of component cell wall parts changes when fungal cells encounter different growth conditions, respond to cell wall stress and undergo morphogenesis is critical to understanding the fungus and deciphering the innate immune responses to this opportunistic fungal pathogen (Vendele et al., 2020).

The main structural components of the *C. albicans* cell wall are chitin (β(1,4)-*N*-acetyl glucosamine) and β(1,3)-glucan and current cell structure models display these skeletal layers as the inner layer of a bilaminate cell wall structure (Bowman and Free, 2006, Gow et al., 2017). Highly mannosylated cell wall proteins (CWPs) are predominantly attached to the structural polysaccharides through glycosylphosphotidylinositol (GPI)-remnants (Gow et al., 2017, Latgé, 2007). The CWPs are decorated with both *N*- and *O*-mannans as they pass through the endoplasmic reticulum and Golgi of the secretory system. The biochemistry of mannosylation is well characterised (Hall and Gow, 2013, Prill et al., 2005). *N*-mannans have a core structure to which outer chain branched *N*-mannan is added. The branches have a long α(1,6)-mannose backbone decorated with α or β mannoside side chains of various lengths. In contrast, *O*-mannan is a simple linear chain made of just a few mannose residues. Both mannan forms can be modified with β(1,2)-phosphomannosides that confer the charged properties of the cell wall (Hall and Gow, 2013).

Based on this information, many different models representing interpretations of the structure of the *C. albicans* cell wall have been published (e.g. Cassone, 1989, Ene et al., 2015, Erwig and Gow, 2016, Free, 2013, Garcia-Rubio et al., 2019, Gow et al., 2017, Gozalbo et al., 1993, Klis et al., 2009, Netea et al., 2008, Osumi, 1998, Ruiz-Herrera et al., 2006, Selitrennikoff, 2001). Most models depict chitin as a thin but distinct layer closest to the cell membrane, a thicker layer of β(1,3)-glucan on top of the chitin, and a superficial outer fibrillar layer of mannosylated CWPs attached to the β(1,3)-glucan via β(1,6)-glucan linkages. However, the nature of very few models has been tested directly and none attempt to describe components of appropriate scalar dimensions. These simplifications have limited our understanding of the design and properties of the cell wall and limited the formulation of emerging hypotheses that can be based on highly abstracted architectures.

Existing models of the cell wall have been based on a combination of methodologies that include: transmission electron microscopy (TEM) and scanning electron microscopy (SEM) examinations that confirm that the outer wall is fibrillar in nature (Osumi, 1998); staining of the wall with various dyes, lectins (Vendele et al., 2020), immune receptor proteins (Graham et al., 2006) and antibodies (Rudkin et al., 2018) with affinity for various polysaccharides and proteins; bioinformatic (Lombard et al., 2014) and proteomic analyses (de Groot et al., 2004, Yin et al., 2008); carbohydrate compositional studies (Klis et al., 2001); nuclear magnetic resonance (NMR) analyses of extracted mannans (Kang et al., 2018, Lowman et al., 2011); and, the examination of ultrastructural comparisons of cell wall mutants (Bruno et al., 2020, Lenardon et al., 2007). Illustrative examples only are listed here. However, the collective insights from these studies have not yet generated an understanding of some fundamental questions such as the biochemical nature of outer mannan rich fibrils, exactly how the major polysaccharides are arranged or where the proteins are located within the laminate wall structure.

In this investigation, we challenged these models of the *C. albicans* cell wall by examining the precise structure, location and molecular sizes of cell wall components using a combination of high pressure freezing/freeze substitution (HPF/FS), TEM, electron tomography and complementary biochemical analyses. Predictions from the emergent cell wall model were informed using a range of glycosylation mutants and enzymatic debridgements that perturb the normal cell wall structure. We present an unbiased three-dimensional (3D) model of the fungal cell wall and a refined scalar model of the *C. albicans* cell wall which is based on cumulative experimental data and molecular scaling factors. This new model can be used to test hypotheses relating to structure–function relationships of the cell wall that underpin the pathobiology of this fungus.

## 2 Materials and methods

### 2.1 Strains and growth conditions

The *C. albicans* strains used in this study are listed in Table 1. Except where indicated, *C. albicans* strains were grown over-night in YEPD + uri (1% (w/v) yeast extract, 2% (w/v) mycological peptone, 2% (w/v) D-glucose supplemented with 25 μg/ml uridine) at 30 °C with shaking at 200 rpm before being diluted to an OD_600_ of 0.2 in fresh YEPD + uri and grown to mid-log phase 6 h at 30 °C with shaking at 200 rpm.

**Table 1:** *C. albicans* strains used in this study.

Name	Published name (s)	Genotype	Source
wild type	CAI-4 + CIp10 (NGY152)	*ura3*Δ::λ*imm434*/*ura3*Δ::λ*imm434*, *RPS1*/*RPS1*::CIp10	(Brand et al., 2004)
*och1*Δ	*och1*Δ + CIp10 (NGY357)	*ura3*Δ::λ*imm434*/*ura3*Δ::λ*imm434*, *och1*Δ::*hisG*/*och1*Δ::*hisG*, *RPS1*/*RPS1*::CIp10	(Bates et al., 2006)
*och1*Δ re-integrant	*och1*Δ + CIp10-*OCH1* (NGY358)	*ura3*Δ::λ*imm434*/*ura3*Δ::λ*imm434*, *och1*Δ::*hisG*/*och1*Δ::*hisG*, *RPS1*/*RPS1*::CIp10-*OCH1*	(Bates et al., 2006)
*pmr1*Δ	*pmr1*Δ + CIp10 (NGY355)	*ura3*Δ::λ*imm434*/*ura3*Δ::λ*imm434*, *pmr1*Δ::*hisG*/*pmr1*Δ::*hisG*, *RPS1*/*RPS1*::CIp10	(Bates et al., 2005)
*pmr1*Δ re-integrant	*pmr1*Δ + CIp10-*PMR1* (NGY356)	*ura3*Δ::λ*imm434*/*ura3*Δ::λ*imm434*, *pmr1*Δ::*hisG*/*pmr1*Δ::*hisG*, *RPS1*/*RPS1*::CIp10-*PMR1*	(Bates et al., 2005)
*chs*3Δ	myco3	*ura3*Δ::λ*imm434*/*ura3*Δ::λ*imm434*, *chs3-2*::*hisG*/*chs3-3*::*hisG-URA3-hisG*	(Bulawa et al., 1995)
*mnt1-mnt2*Δ	NGY112	*ura3*Δ::λ*imm434*/*ura3*Δ::λ*imm434*, *mnt1-mnt2*Δ*::hisG/mnt1-mnt2*Δ*::hisG*	(Munro et al., 2005)
UC820	UC820 (ATCC MYA-3573)	clinical isolate	(Lehrer and Cline, 1969)

### 2.2 Construction, expression and purification of the chitin binding reporter

The chitin binding reporter (ChBD-reporter) was constructed by fusing the chitin binding domain (ChBD) of *Bacillus circulans* chitinase A1 to the human kappa light chain (HuCκ) and a 6xHis tag. A dsDNA gBlocks™ Gene Fragment containing the ChBD (GenBank M57601.1, nucleotides 2183-2338) flanked by 30 bp of non-related sequence and an *Nco*I and *Not*I site was purchased from Integrated DNA Technologies (Table 2), cut with *Nco*I and *Not*I, ligated into the *Nco*I and *Not*I sites of pIMS147 (Hayhurst and Harris, 1999) and transformed into *E. coli* XL1-Blue supercompetent cells (Agilent Technologies). Plasmids were extracted and digested with *Mfe*I which cuts the ChBD but not pIMS147 to confirm the insertion of the ChBD into the vector. Correct integration was confirmed by DNA sequencing using the primer MDL256 (Table 2). The resulting plasmid was designated pChBDHuCκ6xHis.

**Table 2:** DNA fragments and oligonucleotide primers used in this study.

Name	Sequence (5′-3′)	Source
ChBD gBlocks™ (ds DNA)	GGAGTGACTAGCTACTAGACGTGAGACACACCAACCGGCCATGGCCACGACAAATCCTGGTGTATCCGCTTGGCAGGTCAACACAGCTTATACTGCGGGACAATTGGTCACATATAACGGCAAGACGTATAAATGTTTGCAGCCCCACACCTCCTTGGCAGGATGGGAACCATCCAACGTTCCTGCCTTGTGGCAGCTTCAAGCGGCCGCTGCACCATCTGGGCAGCCGCACGGGTCTCGTTTCTCGT	Integrated DNA Technologies
MDL256	CGTATAATGTGTGGAATTGTGAGCG	Invitrogen

The ChBD-reporter was expressed in IPTG-treated XL1-Blue + pChBDHuCκ6xHis cells and purified via the C-terminal 6xHis tag using Ni^2+^ charged immobilised metal ion chelate affinity chromatography (McElhiney et al., 2000). The purified protein was dialysed against PBS at 4 °C and stored in aliquots at −20 °C. Protein concentration in the fractions was estimated using the method described by Bradford (Bradford, 1976) with BSA as a standard. Western blots were performed using monoclonal HuCκ clone KP-53 antibodies (Sigma K4377) to confirm the expression and purification of the ChBD-reporter.

### 2.3 High pressure freezing, freeze substitution and transmission electron microscopy

*C. albicans* strains were grown in YEPD + uri to mid-log phase 6 h at 30 °C. Samples were collected by centrifugation, transferred to specimen carriers and frozen in liquid nitrogen at high pressure using a Leica EM PACT2 high-pressure freezer and EM RTS rapid transfer system (Leica Microsystems). Cells that were treated with enzymes were grown as above, collected by centrifugation, washed in dH_2_O, resuspended in an equal volume of 2x reaction buffer and incubated ± enzyme for 2 h at 37 °C. Samples were then collected by centrifugation, transferred to specimen carriers and frozen under high pressure as above. Enzyme treatments were performed in 100 μl reaction volumes, and the final concentration of enzymes and 1x reaction buffers used were: α(1–2,3,6)-mannosidase from Jack Bean (7.5 mU/μl; GLYKO®) in reaction buffer (20 mM sodium acetate, 0.4 mM Zn^2+^, pH 5.0); α(1–2)-mannosidase from *Aspergillus saitoi* (2 μU/μl; GLYKO®) in reaction buffer (100 mM sodium acetate pH 5.0); pronase from *Streptomyces griseus* (2 μg/μl; Roche) in reaction buffer (0.1 M Tris-HCl pH 7.5, 0.5% SDS); and Endoglycosidase H (Endo H; 5 U/μl; NEB) in G5 reaction buffer (50 mM sodium citrate pH 5.5).

Freeze-substitution was carried out using a Leica EM AFS2 automatic freeze substitution system and EM FSP freeze substitution processor (Leica Microsystems). For samples that were embedded in acrylic resin, freeze-substitution was carried out by dehydrating in dried acetone containing 1% OsO_4_ for 10 h, sequentially warming to −30 °C over 8 h in acetone/OsO_4_, to 20 °C over 3 h in acetone, and then embedding in increasing amounts of Spurr’s (epoxy) resin over 24 h. For samples that were embedded in non-acrylic resin (for gold staining), freeze-substitution was carried out by dehydrating in dried acetone for 24 h at −90 °C, warming to −50 °C over 8 h in acetone, holding at −50 °C for 24 h in acetone, dehydrating in 100% ethanol at −50 °C, embedding in increasing amounts of Lowicryl HM20 resin at −50 °C over 8 h, holding at −50 °C for 24 h, warming to 20 °C over 14 h and holding at 20 °C for 48 h.

Ultrathin sections (90–100 nm) were cut with a diamond knife (Diatome Ltd.) using a Leica UC6 ultramicrotome (Leica Microsystems) onto copper grids (300 mesh), stained with either uranyl acetate and lead citrate or colloidal gold (Section 2.4) and imaged using a Philips CM10 transmission electron microscope (FEI). Images were recorded using Digital Micrograph software (Gatan Inc.).

### 2.4 Colloidal gold staining

The ChBD-reporter (Section 2.2) was used to stain chitin. Ultra-thin sections on copper grids were stained by floating the grids through drops of PBS + 0.02% glycine for 10 min, PBS + 1% BSA for 5 min, ChBD-reporter (20 μg/ml) in PBS + 1% BSA for 1 h in the dark, PBS + 1% BSA 5 × 1 min, anti-HuCκ (raised in mouse, Sigma K4377, 1/50 dilution) in PBS + 1% BSA for 1 h in the dark, PBS + 1% BSA 5 × 1 min, gold-labelled anti-mouse IgG (raised in goat, 40 nm gold particle, KPL #57–18-06, 1/50 dilution) in PBS + 1% BSA for 30 min in the dark, PBS + 1% BSA 4 × 5 min and lastly transferred through 5 drops of dH_2_O. Grids were dried and then stained with uranyl acetate and lead citrate before imaging in the TEM. Competition with chitin was performed by mixing the ChBD-reporter with chitin (5 × chitin: 20 μg/ml ChBD-reporter, 100 μg/ml chitin in PBS + 1% BSA; 10 × chitin: 20 μg/ml ChBD-reporter, 200 μg/ml chitin in PBS + 1% BSA) and incubating for 1 h at RT before staining as described above but using the ChBD-reporter-chitin mixture instead of ChBD-reporter only. Chitin purified from *C. albicans* cell walls was provided by J. Wagener (as described in Wagener et al., 2014). An addition control was performed by staining sections with an unrelated scAb (Hap2) which has affinity for acyl-homoserine lactone signalling molecules produced by bacteria such as *Pseudomonas aeruginosa* (Hayhurst and Harris, 1999).

Fc-dectin-1 (Graham et al., 2006) was provided by G. Brown (University of Aberdeen, now University of Exeter) and used to stain β(1,3)-glucan. Ultra-thin sections on copper grids were stained by floating the grids through drops of PBS + 0.02% glycine for 10 min, PBS + 1% BSA for 5 min, Fc-dectin-1 (10 μg/ml) in PBS + 1% BSA for 1 h in the dark, PBS + 1% BSA 5 × 1 min, gold-labelled anti-human IgG (raised in goat, 40 nm gold particle, KPL #57–10-06, 1/50 dilution) in PBS + 1% BSA for 30 min in the dark, PBS + 1% BSA 4 × 5 min and lastly transferred through 5 drops of dH_2_O. Grids were dried and then stained with uranyl acetate and lead citrate before imaging in the TEM. A gold-labelled antibody-only control was performed as above but excluding the incubation in the presence of Fc-dectin-1.

An anti-mannan single chain antibody fragment (scAb) (Porter et al., 2014) was provided by A. Saxena (Scottish Biologics Facility, University of Aberdeen) and was used to stain mannans. Ultra-thin sections on copper grids were stained by floating the grids through drops of PBS + 0.02% glycine for 10 min, PBS + 1% BSA for 5 min, anti-mannan scAb (10 μg/ml) in PBS + 1% BSA for 1 h in the dark, PBS + 1% BSA 5 × 1 min, anti-HuCκ (raised in mouse, Sigma K4377, 1/50 dilution) in PBS + 1% BSA for 1 h in the dark, PBS + 1% BSA 5 × 1 min, gold-labelled anti-mouse IgG (raised in goat, 40 nm gold particle, KPL #57–18-06, 1/50 dilution) in PBS + 1% BSA for 30 min in the dark, PBS + 1% BSA 4 × 5 min and lastly transferred through 5 drops of dH_2_O. Grids were dried and then stained with uranyl acetate and lead citrate before imaging in the TEM. Competition with mannan was performed by mixing the scAb with mannan (10 μg/ml scAb, 50 μg/ml mannan in PBS + 1% BSA) and incubating for 1 h at RT before staining as described above but using scAb-mannan mixture instead of scAb only. A mannan only control was also performed using mannan (50 μg/ml in PBS + 1% BSA) instead of the scAb. Mannan purified from *C. albicans* was purchased from NIBSC (Product no. 77/600). A gold-labelled antibody-only control was performed as above but excluding the scAb and anti-HuCκ incubations, and a secondary and gold-labelled antibody-only control was performed as above but excluding the incubation in the presence of the scAb.

### 2.5 Gold counts

The distribution mannan, β(1,3)-glucan and chitin was manually assessed by scoring the location of gold particles binding to the cell wall of *C. albicans* in TEM images of sections stained with anti-mannan scAb, Fc-dectin-1 and the ChBD-reporter as described in Section 2.4. The location of the gold particles was scored as binding to either the outer cell wall (fibrillar layer), inner cell wall (between the membrane and fibrillar layer) or directly on the cell membrane. Although the reporter constructs and associated secondary (2°) and tertiary (3°) antibodies that were used in these analyses have a length associated with them (e.g. a typical IgG molecule is 14.5 nm × 8.6 nm × 4 nm (width × length × depth)(Tan et al., 2008), the maximum theoretical length of any tether in these analyses (reporter plus 2° and 3° antibodies) was no more than 25 nm. This was somewhat negated by the 40 nm diameter of the gold particles. Nonetheless, a certain margin of error exists in terms of the precise observed locations of the gold particles on the sections. This was no more than the diameter of a gold particle and the length of the linker was therefore not considered to be a major problem in these analyses. The percentage of the total gold label bound to each cell wall location across sections of cell walls visible in multiple TEM images for each reporter provides an estimate of the distribution. A crude estimate of the average number of gold particles that bound to the cell wall was calculated by dividing the total number of gold particles bound to the wall for each reporter by the number of cell walls visualised across multiple TEM images. On average, the number of images analysed for each condition was 10 or more (range 4 – 34) which were taken from two independent experiments (range 1 – 3).

### 2.6 Electron tomography

*C. albicans* strain wild type strain UC820 (Table 1) was cultured overnight in YEPD + uri broth at 30 °C with shaking at 200 rpm. The culture was diluted in fresh YEPD + uri to an OD_600_ of 0.2 and incubated for 4 h at 30 °C with shaking at 200 rpm. Cells were frozen under high pressure, freeze substituted and embedded in Spurr’s resin as described in Section 2.3. Thick sections (200 nm) were transferred onto 200 mesh copper grips and stained with uranyl acetate and lead citrate. A single axis tilt series of a region of the *C. albicans* cell wall was captured between ± 65° at 1° intervals using a JEM1400 TEM (Jeol Ltd.) set at 120 kV and equipped with a bottom mounted Gatan SC1000 ORIUS CCD camera (Gatan).

The TEM images were compiled into a single axis tomogram using the eTomo interface of IMOD v4.7 image processing package freely available from http://bio3d.colorado.edu/imod/ (Kremer et al., 1996). The full tomogram, reconstructed using the back-projection method, comprised of 2672 × 190 × 2672 slices along the x, y and z axes, respectively. This was further trimmed and realigned to eliminate out of focus slices from the edge of the tilt series. The final tomogram used for modelling comprised of 1545 × 693 × 77 slices, with a voxel resolution of 0.272 nm^3^.

### 2.7 Computer vision and data modelling

Avizo Standard v8.1.1 (FEI) was used for computer vision, image processing and model reconstruction. The computer vision technique of threshold segmentation was used to dissect out microscopic details of the cell wall from the tomogram. During the thresholding process, each pixel in the greyscale image was assigned as either black (0) or white (1) based on a pixel intensity threshold. The optimum pixel intensity threshold was determined using a robust histogram-based thresholding algorithm where the entire spectrum of pixel intensities in an image were plotted and used to detect peaks and minima. The optimum threshold was assigned at the minima. The tomogram was then segmented across all slices using the optimum threshold, which separated out regions of interest from the background and clustered voxels in the regions of interest into 3D volumes. Each component was added as a new “material” in the segmentation set. Isolated segments, known as “islands”, smaller than 15 voxels were either merged or removed using an n-neighbour algorithm. Islands were merged with the neighbour sharing the largest border if it exceeded 25% of the perimeter. Segments were smoothed using an edge-preserving smoothing algorithm (Weickert et al., 1998). The segmented tomogram was used to generate a triangular surface grid using the marching cubes algorithm (Lorensen and Cline, 1987) and unconstrained surface smoothing (smoothing extent: 5). No surface simplification of the triangular mesh was used to avoid data loss which was important for accurate measurements during subsequent model analyses. The triangular mesh was rendered into 3D models using the “opaque-shaded” draw setting.

Analyses of the 3D models were carried out in Avizo Fire v8.1.1 (FEI). The scale was adjusted to nm. Analysis variables included the cell wall dimensions, and total length, branch length, inter-branch length and branch angles of mannan fibrils. The tomogram was divided into 10 sectors and each sector was segmented, modelled and analysed as per the workflow optimised above.

### 2.8 Statistical analyses

Measurements from Avizo Fire were exported into Microsoft Excel 2013 and analysed in SPSS v21.0. Statistical analysis included normality tests and descriptive analysis. Results were reported as means and standard deviations. In addition to deterministic statistical analyses, Monte Carlo stochastic analyses were also performed because only one TEM specimen of a section of *C. albicans* cell wall was used in this study which magnifies the risk of uncertainty errors. Palisade @Risk v5.5 was used for Monte Carlo analysis. In brief, data structure and distribution of each cell wall variable were identified and encoded as input. The final averages of each variable were encoded as output. Each variable was simulated for 5000 iterations and final results were derived from probability histograms.

### 2.9 Modelling of cell wall proteins

The Protein Data Bank (rcsb.org; Berman et al., 2000) was searched for *Candida albicans* protein structures. In addition, structural models for Endo1 and chitin synthase 1 CHS1 (transmembrane and catalytic domain) and chitinase CHT were generated using RaptorX (Källberg et al., 2012). All structural models were visualised in PyMol (The PyMOL Molecular Graphics System, Version 2.0 Schrödinger, LLC.). Specific protein structures or domains displayed include Als3 (PDB ID: 4LEB; Lin et al., 2014), Als9-2 (PDB ID: 2Y7O; Salgado et al., 2011), Sod5 (PDB ID: 4N3T; Gleason et al., 2014), Sap1 (PDB ID: 2QZW; Borelli et al., 2008), Sap3 (PDB ID: 2H6S; Borelli et al., 2007), *endo*-glucanase (PDB ID: 4 K35; Cheng et al., 2009) and *exo*-glucanase (PDB ID: 1EQP; Cutfield et al., 2000). The structure of a chitinase was modelled on the basis of PDB ID: 2UY2 (Hurtado-Guerrero and van Aalten, 2007), and the structure of a Pir protein was modelled based on PDB IDs 4ZOTA, 4AN6A, 2DREA, 4IHZA, 6JBPB (domain 2) and 4BXPA (domain 1). The structure of a cellulose synthase subunit (PDB ID: 4HG6; Morgan et al., 2013) was utilised as an approximation of a chitin synthase. All proteins are depicted in surface representation and have been drawn to scale.

## 3 Results

### 3.1 An unbiased, 3D scalar model of the cell wall informed by electron tomography

The ultrastructure of the *C. albicans* cell wall is preserved extremely well in samples prepared for TEM by HPF/FS compared to conventional methods such as chemical fixation (Fig. S1) allowing measurements at the molecular scale to be made. Transmission electron micrographs of ultrathin sections of *C. albicans* cells imaged in two dimensions (2D) have provided useful information relating to the cell wall architecture. For example, it is clear that the cell wall has two distinct layers, an inner cell wall layer adjacent to the cell membrane, as well as an outer fibrillar layer (e.g. Fig. S1A), and relatively accurate measurements of the thickness of each cell wall layer can be made (e.g. Cheng et al., 2011, Childers et al., 2016, Hall et al., 2013, Sherrington et al., 2017).

**Fig. 1:**
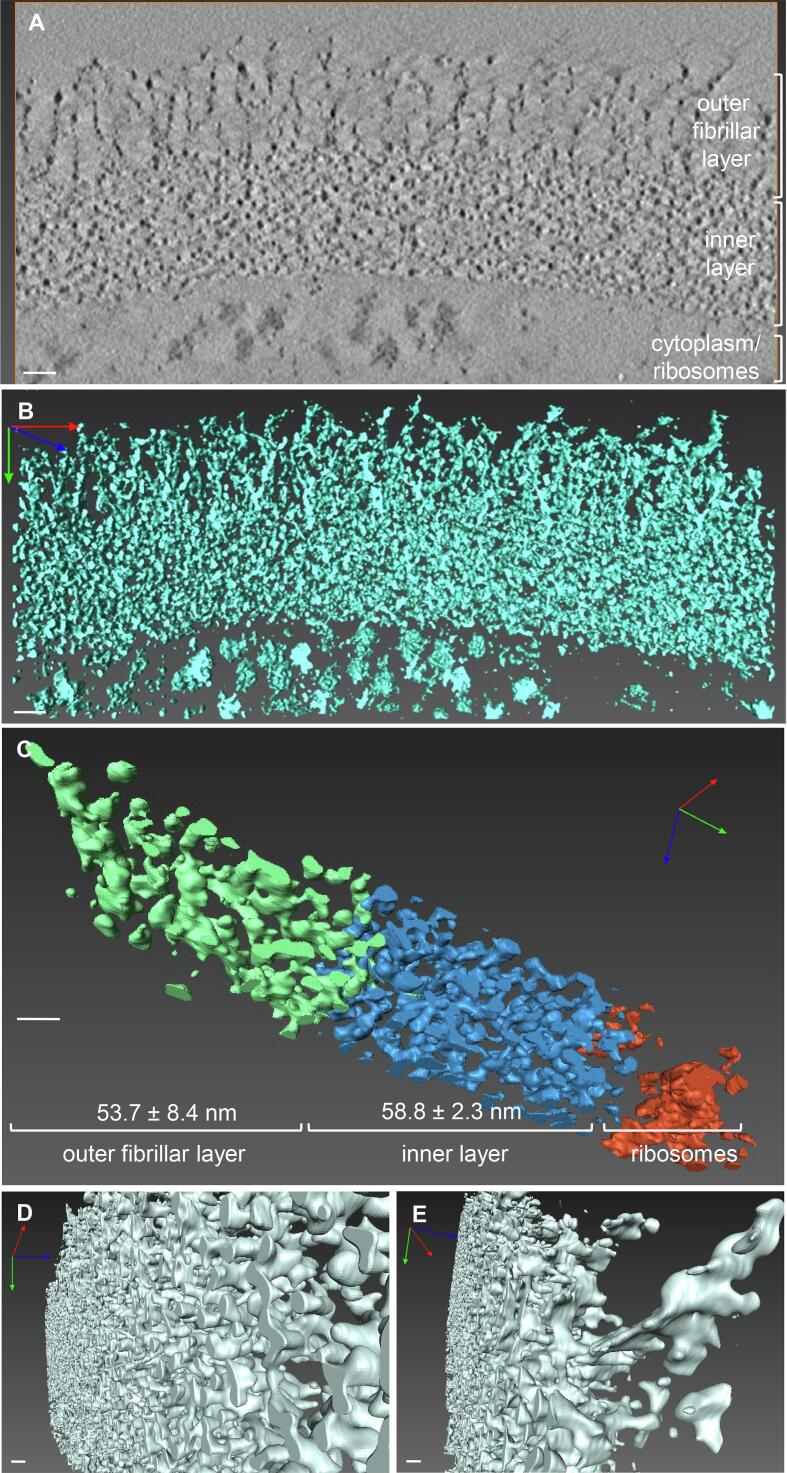
An unbiased 3D model of the *C. albicans* cell wall and outer fibrils. A. A single, 2D slice from the optimised tomogram. The distinct layers of the cell wall are indicated. **B.** Projection of the reconstructed 3D model of the cell wall. The process for generation of the unbiased 3D model (panel B) from the tomogram (panel A) is shown in video 2. **C.** A sector of the cell wall model. The layers have been segmented out individually and rendered in different colours, as indicated. **D**. The inner layer of the wall is a dense but regular, highly interconnected network of fibrils (shown in 3D in video 3). **E.** Fibril shafts decorated with multiple fan-like lateral branches form the outer fibrillary layer of the cell wall. In all panels, the scale bars represent 20 nm. For 3D images, the x, y and z axes are shown in red, green and blue.

To examine the cell wall ultrastructure more precisely, we utilised electron tomography to visualise cell wall ultrastructure in 3D. A high resolution tilt series of a section the cell wall of a wild type *C. albicans* yeast strain was obtained (video 1) and from this, an optimised electron tomogram was generated (1545 nm × 693 nm × 77 slices (x, y, z) with a resolution of 0.272 nm^3^). A single slice of the tomogram is illustrated in Fig. 1A. An unbiased 3D model was then generated from the tomogram using the computer vision technique of threshold segmentation (Fig. 1B; video 2).

The outer fibrillar layer and inner layer of the cell wall were clearly distinguishable in the 3D cell wall model (Fig. 1B). To make accurate measurements of the thickness of each layer, the model was divided into 10 smaller sectors, and the distinct layers of the cell wall were segmented out and rendered in different colours (Fig. 1C). The thickness of each cell wall layer was calculated from measurements made at 12 evenly spaced points across the model. This revealed that the inner layer was uniform in thickness (53.8 ± 2.3 nm (mean ± SD)), whereas the thickness of the outer fibrillar layer was slightly more variable (53.7 ± 8.4 nm) (Fig. 1C).

The organisation of each of the cell wall layers was also visually assessed. The inner layer was made up of a dense but regular network of horizontally arranged fibrils, which was extensively interconnected and was detected as a single 3D volume during segmentation (Fig. 1D; video 3). However, the finer details of the inner cell wall (e.g. CWPs, β(1,3)-glucan vs. β(1,6)-glucan, chitin microfibrils) could not be delineated due to the inability of electron microscopy to distinguish components of similar electron density. The outer fibrillar layer was made up of vertically arranged fibrils which emerged perpendicular to the inner layer (Fig. 1E), with a spiny shaft decorated with multiple lateral branches of variable lengths. This arrangement is consistent with the structure and predicted molecular dimensions of the α(1,6)-mannose backbone and side chains of the *N*-mannans which are attached to CWPs (discussed in Section 3.3).

### 3.2 Chitin is interspersed with β(1,3)-glucan in the inner layer, and mannans comprise the outer fibrillar layer of the cell wall

**Fig. 2:**
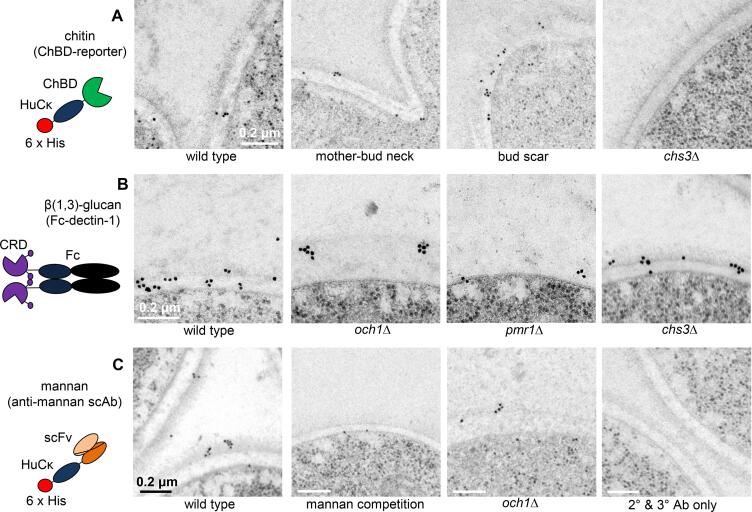
The distribution of chitin, β(1,3)-glucan and mannan in the *C. albicans* cell wall. A. Electron micrographs of wild type (CAI-4 + CIp10) and *chs3*Δ cells stained with the ChBD-reporter/anti-HuCκ Ab/anti-mouse IgG-gold. B. Electron micrographs of sections of wild type (CAI-4 + CIp10), *och1*Δ, *pmr1*Δ and *chs3*Δ cells stained with Fc-dectin-1/anti-human IgG-gold. C. Electron micrographs of wild type (CAI-4 + CIp10) and *och1*Δ cells stained with anti-mannan scAb/anti-HuCκ Ab/anti-mouse IgG-gold. The mannan competition was performed by mixing the scAb with purified mannan before staining sections. The secondary (2°) and tertiary (3°) antibody-only control was performed by staining sections with anti-HuCκ Ab/anti-mouse IgG-gold only. Scale bars represent 0.2 μm and are the same for all images in panels A and B, and as indicated on the images in panel C. Diagrams of the antibodies/reporters are provided to the right of each panel. ChBD – chitin binding domain; HuCκ – human kappa light chain; 6 × His – hexahistidine tag; CRD – carbohydrate recognition domain and stalk region of dectin-1; Fc - Human IgG1 Fc region; scFv – single chain variable fragment (mannan-binding domain).

To determine the precise location of each carbohydrate component in the cell wall, ultrathin sections of *C. albicans* cells embedded in a non-acrylic resin (HM20) were stained with antibodies/reporter constructs and binding was detected using antibodies to these reporters conjugated to colloidal gold (Fig. 2). The ultrastructure of the cell wall was slightly less clear in cells embedded in non-acrylic resin (e.g. HM20) compared to those embedded in acrylic resin (e.g. Spurr’s resin) (compare Fig. 2A to Fig. S1A) but non-acrylic resin gave a lower background of non-specific colloidal gold staining (data not shown).

**Fig. 3:**
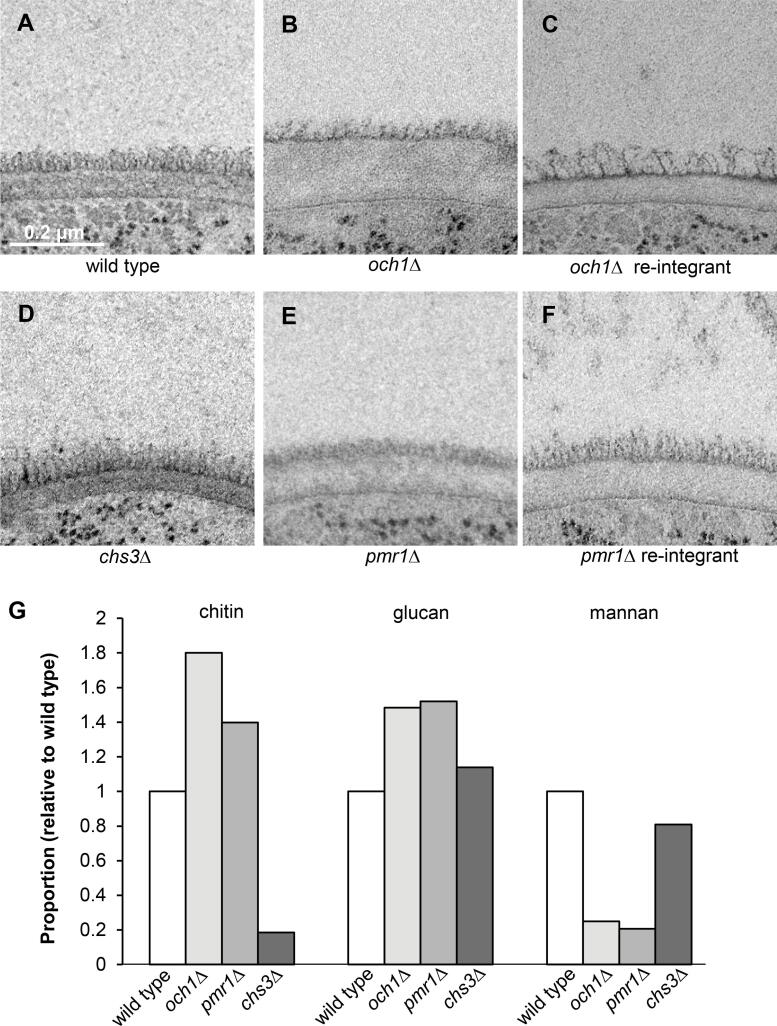
Deletion of genes encoding enzymes that synthesise cell wall components alters the ultrastructure of the *C. albicans* cell wall. Electron micrographs of the cell walls of the: A. wild type (CAI-4 + CIp10); B. *och1*Δ mutant; C. *och1*Δ re-integrant; D. *chs3*Δ mutant; E. *pmr1*Δ mutant; and F. *pmr1*Δ re-integrant strains. The scale bar shown in panel A represents 0.2 μm and is the same for all images. G. The relative proportions of mannan, but not chitin and glucan, in the cell walls of *C. albicans* strains correlate with the changes in cell wall ultrastructure. The amount of *N*-acetylglucosamine (from chitin), glucose (from glucan) and mannose (from mannan) in the cell wall was determined by HPLC using the method described in (Plaine et al., 2008). For the wild type strain, these were 24.8, 533.7 and 281.5 μg/mg dry weight of the wall respectively. The relative proportions shown in the graph were calculated after setting the amount of each sugar in the wall of the wild type cell wall to 1.0 using the data from (Lenardon et al., 2010a).

To stain chitin, a chitin-binding reporter (ChBD-reporter) was constructed consisting of the chitin binding domain (ChBD) of *Bacillus circulans* chitinase A1 fused to the human kappa light chain (HuCκ) (Fig. 2A). In contrast to this ChBD-reporter, use of the colloidal-gold labelled lectin wheat germ agglutinin (WGA) showed a high amount of non-specific binding to resin in samples that had been prepared by HPF/FS (data not shown). A soluble chimeric form of dectin-1 (Fc-dectin-1) consisting of the carbohydrate recognition domain and stalk region of dectin-1 coupled to the Fc region of human IgG1 was used to stain β(1,3)-glucan in the cell wall (Fig. 2B). A single chain antibody (scAb) that specifically recognises *C. albicans* mannan (Porter et al., 2014) was used to determine the location of mannans in the cell wall (Fig. 2C). Binding of the antibodies/reporters to ultrathin sections containing wild type cells, *chs3*Δ mutant cells (which contain 0.18 × of the chitin observed in wild type cells; Fig. 3G), *och1*Δ mutant cells (with 1.8 × of the chitin, 1.5 × glucan and 0.25 × mannan compared to wild type cells; Fig. 6G), and *pmr*1Δ mutant cells (with 1.4 × chitin, 1.5 × glucan and 0.2 × mannan compared to wild type cells; Fig. 3G) was assessed. The distribution of the gold particles in each layer of the cell wall was also assessed semi-quantitatively (Table 3). An example of how the quantification was carried out is provided in Fig. S2.

**Table 3:** Distribution of gold-particles in the layers of the cell wall.

Reporter	*C. albicans* strain	Outer (fibrillar layer)	Inner (between fibrils and membrane)	Cell membrane	(total gold particles counted)	Average gold particles per cell wall*	(cell walls)
ChBD-reporter	wild type	7.4%	78.3%	14.2%	(n = 351)	4.2	(n = 83)
ChBD-reporter + 5x chitin	wild type	31.8%	36.4%	31.8%	(n = 44)	1.4	(n = 32)
ChBD-reporter + 10x chitin	wild type	0.0%	100.0%	0.0%	(n = 1)	0.1	(n = 19)
ChBD-reporter	*chs3*Δ	36.4%	63.6%	0.0%	(n = 11)	0.4	(n = 26)
anti-HuCκ, anti-mouse IgG-gold	wild type	0.0%	0.0%	100.0%	(n = 4)	0.1	(n = 42)
Irrelevant Hap2 scAb	wild type	0.0%	70.0%	30.0%	(n = 10)	0.9	(n = 11)
Fc-dectin-1	wild type	18.5%	64.3%	17.2%	(n = 1151)	14.9	(n = 77)
anti-human IgG-gold	wild type	0.0%	0.0%	0.0%	(n = 0)	0.0	(n = 19)
Fc-dectin-1	*och1*Δ	19.6%	71.0%	9.3%	(n = 535)	24.3	(n = 22)
Fc-dectin-1	*och1*Δ re-integrant	17.4%	64.6%	18.1%	(n = 144)	7.2	(n = 20)
Fc-dectin-1	*pmr1*Δ	24.1%	64.0%	11.9%	(n = 303)	11.2	(n = 27)
Fc-dectin-1	*pmr1*Δ re-integrant	21.2%	59.4%	19.4%	(n = 520)	11.1	(n = 47)
Fc-dectin-1	*chs3*Δ	22.0%	67.5%	10.5%	(n = 909)	33.7	(n = 27)
anti-mannan scAb	wild type	48.2%	21.2%	30.6%	(n = 353)	13.1	(n = 27)
anti-mannan scAb + mannan	wild type	0.0%	8.6%	91.4%	(n = 35)	2.9	(n = 12)
mannan	wild type	0.0%	0.0%	0.0%	(n = 0)	0.0	(n = 11)
anti-mannan scAb	*och1*Δ	20.8%	24.5%	54.7%	(n = 106)	8.2	(n = 13)
anti-mannan scAb	*pmr1*Δ	0.0%	0.0%	100.0%	(n = 5)	0.3	(n = 15)
anti-mouse IgG-gold	wild type	33.3%	66.7%	0.0%	(n = 6)	0.5	(n = 13)

*see Fig. S2 for an example of this calculation.

The chitin-specific ChBD-reporter mostly bound to the inner layer of the cell wall with ~ 78% of the gold label detected on this layer of the cell wall (Table 3). Binding was detected in the cell wall (Fig. 2A left panel), at the mother-bud neck (Fig. 2A middle left panel) and was enriched at bud scars (Fig. 2A middle right panel). The gold particles were distributed throughout the inner layer and not as a distinct sub-layer closest to the cell membrane (Fig. 2A first three panels). Chitin purified from *C. albicans* cell walls was able to compete out the binding of the ChBD-reporter (Table 3) and almost no binding was observed to sections of the *chs3*Δ mutant which has very little chitin in its cell wall (Fig. 2A right panel; Table 3), confirming that this ChBD-reporter was chitin-specific. Furthermore, no gold labelling was observed when sections were stained with the 2° (anti-HuCκ) and 3° (anti-mouse IgG-gold) antibodies only (Fig. 2C right panel; Table 3) and very little binding was observed for an irrelevant scAb (Hap2; Table 3). As an approximation, the density of gold particle labelling of the chitin, glucan and mannan layers reflected the known abundance of each polymer in the cell wall. For example, the amount of gold label detected per wild type cell wall for the ChBD-reporter (average ~ 4 gold particles per section) was much less than for the anti-mannan scAb (average ~ 13) or Fc-dectin-1 (average ~ 15) which is consistent with the relatively low proportion of chitin in the wild type cell wall compared to mannan or glucan (~25 μg/mg dry weight of the cell wall for chitin, ~530 for glucan and ~ 280 for mannan; Fig. 3G).

Fc-dectin-1 bound predominantly to the inner layer of the cell wall of wild type cells (Fig. 2B left panel) with ~ 64% of the gold label detected on this layer of the wall (Table 3). The remainder of the label was roughly equally distributed between the cell membrane (~17%) and the outer cell wall layer (~18%) (Table 3). This is consistent with the presence of the glucan synthase complex in the cell membrane (Frost et al., 1994) from which nascent chains of β(1,3)-glucan presumably protrude, and some β(1,3)-glucan fronds extending into the outer layer of the cell wall (Childers et al., 2020). No gold labelling was observed when sections of wild type cells were stained with the secondary (anti-human IgG-gold) antibody alone (Table 3). A higher proportion of Fc-dectin-1 bound to the inner layer of the cell wall of the *och1*Δ mutant (71%; Table 3) than wild type, and binding tended to be in regularly spaced clusters along the wall (Fig. 2B middle left panel). More Fc-dectin-1 gold label was detected per cell wall of the *och1*Δ mutant compared to wild type (average ~ 24 gold particles per cell wall section compared to ~ 15 gold particles per cell wall; Table 3) which is consistent with there being ~ 1.5 times more glucan in the *och1*Δ cell wall (Fig. 3G). However, the amount of binding to the inner layer of the cell wall of the *pmr1*Δ mutant was similar to that of the wild type (~64%; Table 3) despite there being ~ 1.5 times more glucan in the *pmr1*Δ cell wall compared to wild type (Fig. 3G). Nevertheless, a similar clustered arrangement of binding was observed as for the *och1*Δ mutant (Fig. 2B middle right panel). One possible explanation for the difference is that the β(1,3)-glucan in the inner layer of the cell wall may be arranged differently in the *pmr1*Δ mutant compared to the *och*1Δ mutant making it less accessible for binding by Fc-dectin-1. The binding of Fc-dectin-1 to the inner layer of the cell wall of the *chs3*Δ mutant was similar to that of the wild type in both the amount (~68%; Table 3) and distribution (Fig. 2B right panel).

The anti-mannan scAb predominantly bound to the fibrils of wild type cells (Fig. 2C left panel) with ~ 48% of the gold label detected on this layer of the cell wall (Table 3). However, 21% percent of the gold label was detected on the inner layer of the cell wall and ~ 30% on the cell membrane (Table 3) suggesting that mannosylated CWPs are also embedded deeper in the cell wall and/or cell membrane. Free mannan was able to compete out the binding of this scAb to the cell wall (Fig. 2C middle left panel; Table 3) confirming that the scAb was mannan-specific. A small amount of binding to the outer layer of short fibrils was observed for the *och*1Δ mutant (Fig. 2C middle right panel; Table 3) but not to the fibrils of the *pmr1*Δ mutant (Table 3). Almost no non-specific gold labelling was observed when sections were stained with the gold labelled antibody (anti-mouse IgG-gold) only (Table 3) and no gold labelling was observed when sections were stained with 2° and 3° antibodies only (Fig. 2C right panel; Table 3).

These results indicate that the arrangement of chitin is not consistent with current cell wall models in the literature that portray chitin as a discreet innermost layer close to the cell membrane. Instead, chitin is interspersed with the β(1,3)-glucan in the inner layer of the cell wall. β(1,3)-glucan is more abundant than chitin and is dispersed throughout the inner layer of the cell wall of the wild type strain (and *chs3*Δ mutant strain) but tends to be more heterogeneous in density in the *och1*Δ and *pmr1*Δ mutant strains. The molecular organisation of the β(1,3)-glucan in these mutants must also differ as the β(1,3)-glucan in the *och1*Δ mutant is more accessible to binding by Fc-dectin-1 than in the *pmr1*Δ mutant. These results support the idea that most of the mannans on mannosylated CWPs radiate out from the cell wall to form the outer fibrillar layer. However, some may be embedded deeper in the cell wall and cell membrane.

### 3.3 Cell wall proteins are dispersed throughout the inner layer of the cell wall

**Fig. 4:**
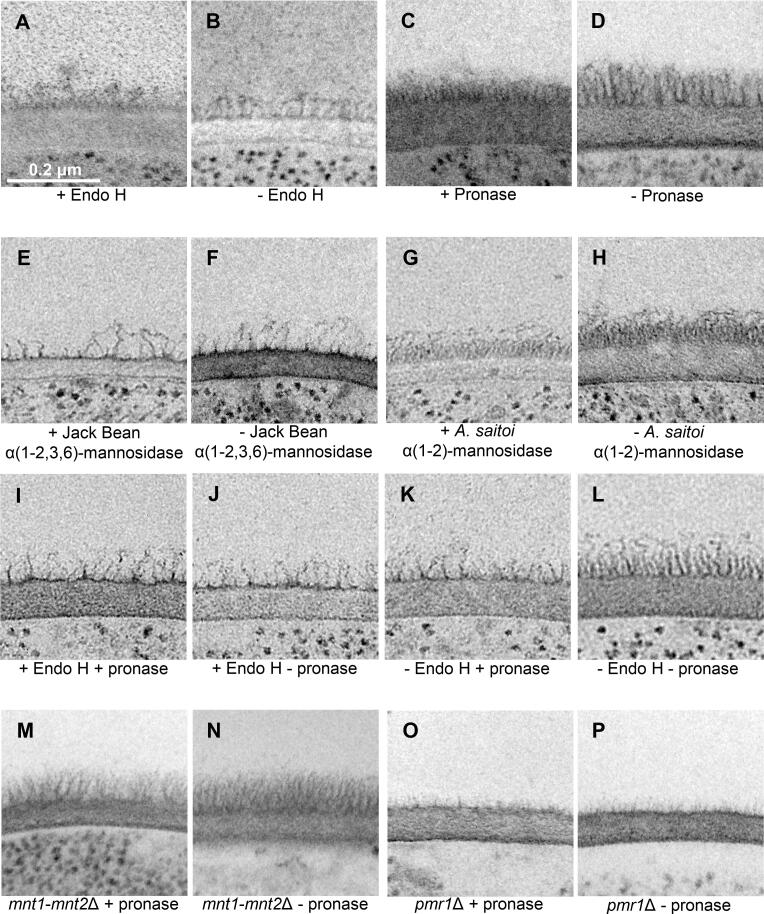
Treatment with enzymes that degrade cell wall components alters the *C. albicans* cell wall ultrastructure. A-H. Electron micrographs of cell walls of wild type (CAI-4 + CIp10) cells treated with (+) and without (-) enzyme for 2 h at 37 °C. ± Endo H (A, B), ± pronase (C, D), ± α(1–2,3,6)-mannosidase from Jack Bean (E, F), ± α(1–2)-mannosidase from *Aspergillus saitoi* (G, H). I-L. Electron micrographs of cell walls of wild type (CAI-4 + CIp10) cells treated sequentially with (+) and without (-) Endo H then with (+) and without (-) pronase for 2 h at 37 °C. M−P. Electron micrographs of cell walls of mutant strains treated with (+) and without (-) pronase for 2 h at 37 °C. *mnt1*-*mnt2*Δ ± pronase (M, N), *pmr1*Δ mutant ± pronase (O, P). The scale bars represent 0.2 μm and are the same for all panels.

To probe the arrangement of mannosylated CWPs in the cell wall in more detail, we examined the effect of treating cells with enzymes that degrade mannans and proteins on the ultrastructure of the cell wall. Exponentially growing yeast cells were treated with Endoglycosidase H (Endo H) to remove high molecular weight *N*-glycans from glycoproteins, pronase to degrade cell wall proteins, and α(1-2,3,6)-mannosidase from Jack Bean and α(1-2)-mannosidase from *Aspergillus saitoi* to remove specific mannan residues. The cells were then prepared for TEM by HPF/FS. The cell wall ultrastructure of cells treated with enzymes for 2 h at 37 °C was compared to that for cells incubated in buffer only (Fig. 4A–H).

Fibrils radiating out of the cell wall were still present on cells that had been exposed to Endo H (Fig. 4A) but were shorter than those on cells incubated in buffer only (Fig. 4B) and material blebbing off the cell wall was observed. The fibrils on cells treated with pronase (Fig. 4C) were approximately half the length of those incubated in buffer alone (Fig. 4D), and individual fibrils were less easy to distinguish. Treatment with α(1-2,3,6)-mannosidase from Jack Bean (Fig. 4E) appeared to have a major destructive effect on the architecture of the fibrils radiating out of the cell wall compared to cells incubated in buffer only (Fig. 4F). Most fibrils were almost entirely removed, but some were shortened or disordered. Treatment of cells with α(1-2)-mannosidase from *A. saitoi* (Fig. 4G) had little effect on the ultrastructure of the cell wall compared to cells incubated in buffer only (Fig. 4H).

Taken together, these observations indicate that the fibrils radiating out from the cell wall are predominantly represented by the outer chains of the *N*-mannans that are amide-linked to CWPs. Endo H and α(1-2,3,6)-mannosidase from Jack Bean, which degrades linkages found in the *N*-mannan backbone, caused a reduction in the length of the fibrils. In contrast, most α(1,2)-mannans form the short, linear *O*-mannan chain and decorate the branches of the *N*-mannan chains that emanate from the α(1,6)-*N*-mannan backbone. Thus activity is consistent with lack of impact of treatment with α(1–2)-mannosidase from *A. saitoi* upon the overall length of the fibrils. The fractional reduction in the length of some of the fibrils upon treatment with pronase may indicate that only those cell wall proteins that are displayed right on the very top of the chitin and β(1,3)-glucan layer were susceptible to digestion, but those embedded deeper in this layer were not, perhaps because they were not accessible to pronase. An alternative interpretation is that the mannan structures partially protect the CWPs from digestion with pronase.

To differentiate between these two possibilities, the mannans were first removed from cells, then treated with pronase, and then prepared for TEM analysis by HPF/FS (Fig. 4I-P). *N*-mannans were removed from cells by treatment with Endo H for 2 h at 37 °C, treated with pronase for a further 2 h at 37 °C (Fig. 4I) and the ultrastructure of the cell wall was compared to that from cells treated with only one enzyme (Fig. 4J, K) and a buffer only control (Fig. 4L). The cells of an *mnt1-mnt2*Δ mutant which has no *O*-mannan chains (Munro et al., 2005) and a *pmr1*Δ mutant which is deficient in *N*-mannan, phospho-mannan and has a severe *N*- and *O*-glycosylation defect (Bates et al., 2005) were also treated with pronase for 2 h at 37 °C (Fig. 4M, O) and compared to buffer only controls (Fig. 4N, P).

Sequential treatment of cells with Endo H and then pronase (Fig. 4I) did not result in additional fibrils being removed or shortened compared to cells treated with pronase alone (Fig. 4K). Only a slight disordering of the fibrils was observed in cells of the *mnt1-mnt2*Δ mutant and *pmr*1Δ mutant that had been treated with pronase (Fig. 4M, O) compared to cells incubated in buffer only (Fig. 4N, P). This is consistent with the known effects of *O*-glycosylation stabilising the rod-like domains of typical of serine-threonine rich regions of glycoproteins (Frieman et al., 2002). This suggests that mannans do not protect the CWPs from digestion with pronase and therefore supports the idea that the CWPs that are embedded deeper in the chitin and β(1,3)-glucan layer are less accessible to pronase.

### 3.4 Modelling of individual mannan fibrils reveals the scalar architecture of *C. albicans N*-mannan

Having determined that the outer fibrillar layer of the wild type cell wall is predominantly *N*-mannan, we investigated the 3D structure of the *N*-mannan fibrils in detail. Single fibrils were analysed in our 3D cell wall model which was generated from the cell wall tomogram (Fig. 1B; video 2) and modelled individually (video 4). An example of an intact and discrete fibril is shown in video 5 and Fig. S3A. Of 16 fibrils examined in detail, a variable total length ranging from 36.8−96.7 nm with a mean of 65.5 nm (SD: ± 4.0 nm), calculated using Monte Carlo statistics (Section 2.8), was observed (Fig. S3A). These values are compatible with published reports (Gow et al., 2017, Osumi, 1998).

The mannan fibrils modelled above were decorated with multiple lateral branches of varying lengths. Lateral branches were measured for their 3D length (Fig. S3B) and were found to vary between 5.3 and 22.8 nm (n = 86). The mean branch length computed by the Monte Carlo algorithm was 10.4 nm (SD: ± 0.3 nm). The lateral branches on the mannan fibrils were separated from each other by a regular 3D distance. This inter-branch length was measured between pairs of successive lateral branches (Fig. S3C). Statistical analysis showed that this inter-branch length was remarkably regular, varying between 3.9 and 6.8 nm, with a mean of 5.1 nm (SD: ± 0.1 nm; n = 89).

The angles at which the lateral branches emerged from the spine were also reasonably uniformly distributed. Statistical analysis revealed that majority of these branches emerged nearly perpendicular to the spine. The branch angle varied between 59.0° − 115.9° with the average angle being 94.4° (SD: ± 1.3°, n = 74) (Fig. S3D).

In order to determine the arrangement of the branches around the spine, the models of the individual mannan fibrils were rotated and visualised from the top. For mannan fibrils that had a backbone which was straight enough for analysis to be performed (n = 4), the lateral branches were seen to be arranged one below the other in a plane angulated to the coronal plane of the mannan spine (Fig. S3E). The angulated plane varied between 33.2° and 42.8°.

**Fig. 5:**
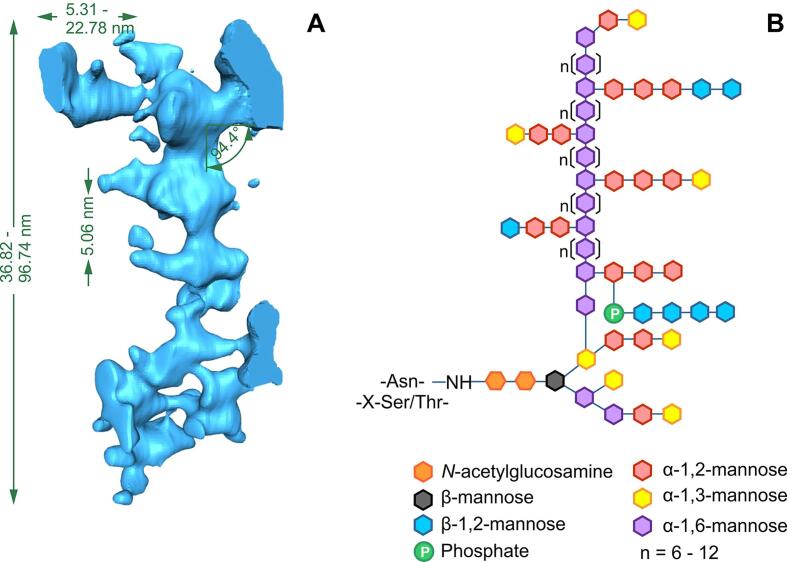
Scalar architecture of *N*-mannan fibrils. A. Dimensions of the *N*-mannan fibrils calculated from 16 discrete and intact mannan fibrils in our 3D cell wall model. The total fibril length (range), branch length (range), inter-branch length (mean) and branch angle (mean) are indicated (see Fig. S3 for explanation of the measurements). B. Scalar architecture of *C. albicans N*-mannan. The α(1,6)-mannose backbone is calculated to be between 74 and 194 mannose residues long, with the side branches varying in length from 10 − 45 mannose residues and emerging from opposite sides of the α(1,6)-backbone every 6–12 (mean: 10) mannose residues. Coloured hexagons represent specific linkages between sugar residues, as indicated.

This information gathered from the measurements of the 3D structure of individual mannan fibrils in our 3D model (Fig. 5A) was then used to develop a scalar architecture for the chemical structure of *C. albicans N*-mannan. Given that the mannopyranose ring is 5 Å or 0.5 nm in diameter, we estimated that the α(1,6)-mannose backbone of the cells grown under the conditions of this analysis were 74–194 mannose residues long. The side branches varied in length from 10 − 45 mannose residues and emerge from opposite sides of the α(1,6)-backbone every 6–12 (mean: 10) mannose residues (Fig. 5B).

### 3.5 Correlations between the chemical and physical structure of the cell wall

We then determined whether changes in the relative amounts of the major cell wall polysaccharides correlated with changes in the observed wall architecture by examining the effect of deleting genes encoding enzymes that synthesise key cell wall components on the observed ultrastructure of the cell wall.

The cell walls of exponentially growing *C. albicans* yeast cells that had been prepared for TEM by HPF/FS were imaged. The ultrastructure of the cell wall of a wild type strain was compared to that of an *och1*Δ mutant which has a severe *N*-glycosylation defect (Bates et al., 2006), a *chs*3Δ mutant which has very little chitin its cell wall (Bulawa et al., 1995) and a *pmr1*Δ mutant which has a severe *N*- and *O*-glycosylation defect (Bates et al., 2005). Our observations were correlated with the known proportions of cell wall carbohydrates in these strains relative to the wild type (Fig. 3).

The cell wall of the wild type strain consisted of two layers, an inner layer approximately 60 nm wide immediately adjacent to the cell membrane, and an outer fibrillar layer approximately 40–45 nm wide (Fig. 3A). In the *och1*Δ mutant (Fig. 3B), the fibrils radiating out of the cell wall were shorter (~28 nm) and more disordered compared to the wild type and *och1*Δ re-integrant strains (Fig. 3A, C), and the *och1*Δ mutant had four times less mannan in its cell wall compared to the wild type strain (Fig. 3G). The inner layer of the cell wall of the *och1*Δ mutant was approximately three times thicker (~140 nm) than that of the wild type strain (Fig. 3A), which correlated with an increase in the proportions of chitin (~1.8 times more) and glucan (~1.5 times more) in the cell wall of the *och*1Δ mutant compared to the wild type strain (Fig. 3G).

The *chs*3Δ mutant (Fig. 3D) showed no gross alterations in the ultrastructure of the cell wall compared to the wild type strain (Fig. 3A), despite there being approximately five times less chitin in the cell wall compared to the wild type strain (Fig. 3G). The *pmr1*Δ mutant (Fig. 3E) had shorter fibrils compared to the wild type and re-integrant strains (Fig. 3A, F) and almost five times less mannan in the cell wall of this strain compared to the wild type strain (Fig. 3G). However, the thickness of the inner layer of the cell wall was only slightly increased (~78 nm) compared to the wild type strain, despite there being ~ 1.4 times more chitin and ~ 1.5 times more glucan in the cell wall of this strain compared to the wild type strain (Fig. 3G).

Taken together, these observations further support the idea that the fibrils that radiate out of the cell wall are *N*-mannan outer chains that are attached to CWPs and that the inner layer is comprised of β(1,3)-glucan interspersed with chitin. The reduction in the length of the *N*-mannan fibrils of the *och1*Δ and *pmr1*Δ mutants correlated well with the reduction in the amount of mannan in the cell wall. However, the contribution of the amount of chitin and β(1,3)-glucan to the overall thickness of the inner layer of the cell wall was less clear: a 5-fold reduction in the amount of chitin the cell wall had little effect of the overall thickness of the inner layer of the cell wall (*chs3*Δ); increasing the amount of β(1,3)-glucan 1.5-fold and chitin by 1.4-fold had little effect on the thickness of the cell wall (*pmr1*Δ); however, the same increase in the amount of β(1,3)-glucan accompanied by a 1.8-fold increase in chitin (*och1*Δ) resulted in a tripling of the thickness compared to wild type. This may indicate that the thickness of the inner layer of the cell wall is not determined by the gross levels of chitin and β(1,3)-glucan, but rather by the 3D organisation of these polysaccharides. This suggests that other characteristics of the *och1*Δ and *pmr1*Δ mutants (e.g. the CWPs linked to the chitin and β(1,3)-glucan) result, either directly or indirectly, in alterations to the organisation of the chitin and β(1,3)-glucan layer which affect the thickness of this layer of the cell wall.

## 4 Discussion

**Fig. 6:**
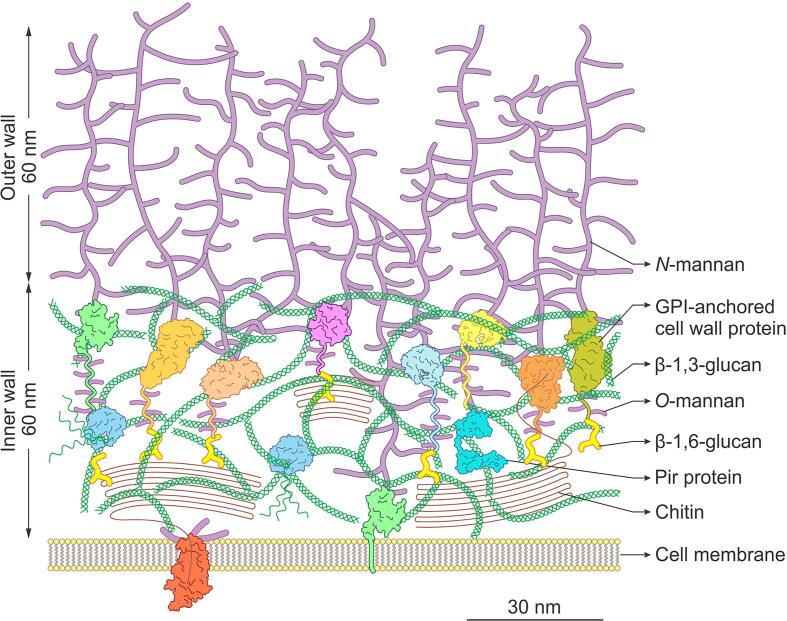
Refined, to-scale model of the *C. albicans* cell wall. The inner layer of the cell wall is comprised of chitin microfibrils (brown) interspersed with triple and single helices of β(1,3)-glucan (green). Mannosylated cell wall proteins are distributed throughout the inner cell wall layer, with a small proportion in the cell membrane. The *N*-mannan structures (purple) attached to cell wall proteins form the outer fibrillar layer of the cell wall. GPI-anchored cell wall proteins are abundant and have a globular domain to which long *N*-mannan structures are attached, and a stalk region to which short *O*-mannans (purple) are attached. Examples shown in the figure are Als3 (chrome yellow), Als9-2 (light olive green), Sod5 (mint green), Sap1 (lemon yellow) and Sap3 (sky blue), an *endo*-glucanase (beige), an *exo*-glucanase (pink) and a chitinase (orange). These are covalently linked to chitin and β(1,3)-glucan via β(1,6)-glucan linkages (yellow). Pir cell wall proteins (turquoise) are less abundant and are *N*-mannosylated and covalently linked to β(1,3)-glucan via an unknown alkali-sensitive bond. A partial chitin synthase enzyme with its catalytic core and transmembrane domain is depicted in the membrane (red). The scale bar represents 30 nm.

Through in-depth, high resolution transmission electron microscopy and electron tomography analyses, new information has been revealed about the precise structure and architecture of the *C. albicans* cell wall. This enabled a physical and chemical interpretation of the outer cell wall fibrils to be made for the first time. We suggest that these fibrils represent the outer chain *N*-mannan comprising the α(1,6)-linked mannose backbone and subtended sidechains of α(1,2)-, α(1,3)-, β(1,2)-mannan and phosphomannan. The information provided also challenges generally accepted models of the *C. albicans* cell wall in a number of respects: (i) it challenges the view that chitin is distributed as a distinct layer next to the membrane and suggests that it is distributed throughout the inner cell wall layer; (ii) it demonstrates that *N*-mannans form the outer fibrillar layer of the cell wall that are attached to CWPs which are embedded in wall; and (iii) it suggests that some mannosylated CWPs are distributed throughout the inner layer of the wall, but that there is an enrichment of proteins at the interface of the inner wall and outer fibrillar layer of the wall. This suggests that the long-axis of the fibrils resolved in the outer *C. albicans* wall represents the highly elaborated *N*-mannan outer chain of GPI-anchored glycoproteins and that the polypeptide component of these proteins resides basal to the outer cell wall fibrils. We present a refined model of the *C. albicans* cell wall depicting a section of mature cell wall from a *C. albicans* yeast cell (Fig. 6) that is based on components represented at their true molecular scale. A discussion of the rationale behind the depiction of each cell wall component in the new and refined cell wall model is provided in 4.1, 4.5 below. In addition to our refined model of the mature yeast cell wall, we also have presented icons of the individual components of this new model as part of a “draw your own cell wall” kit (Fig. S4) which we hope will be a useful resource to the community.

### 4.1 Chitin

In *C. albicans,* chitin is found in the form of α-chitin where adjacent chains of the homopolymer are antiparallel and hydrogen bond to form microfibrils (Lenardon et al., 2010b). We have represented chitin in its microfibrillar form in the refined model. The size of the microfibrils depicted is based on measurements of chitin microfibrils in chitin ghosts (e.g. Lenardon et al., 2007) and takes into account the dimensions of α-chitin crystals. For example, chitin microfibrils are reported to have a diameter of 9–15 nm and an average length of 33 ± 16 nm (Gow and Gooday, 1983), and a chitin dimer is 10.3 Å (1.03 nm) long and two anti-parallel chains are 18.9 Å (1.89 nm) wide (Sikorski et al., 2009). Therefore, an average chitin microfibril, which is 33 nm long and 12 nm wide, corresponds to antiparallel chains around 64 residues long, with 12 bends.

We depict chitin microfibrils distributed throughout the inner cell wall layer which is consistent with our staining of chitin with the ChBD-reporter (Fig. 2A), and other TEM analyses which used gold-labelled WGA to stain chitin (Badrane et al., 2012, Marcilla et al., 1991). Because chitin is a linear, non-flexible molecule, a continuous layer of chitin surrounding the cell adjacent to the membrane would render the wall inflexible. This is not consistent with the compliance of the cell wall to osmotic swelling (e.g. Ene et al., 2015). Although we have not included linkages between chitin microfibrils in the refined model, these linkages would need to provide rigidity to the cell wall whilst maintaining the necessary flexibility for cells, e.g., to quickly respond to external stresses, such as when cells expand or contract within seconds following exposure to osmotic stress (Ene et al., 2015). They also need to be able to be compliant to allow liposomes and/or extracellular vesicles, which are much larger than the theoretical porosity of the cell wall, to pass through the wall (e.g. Walker et al., 2018). We therefore hypothesise that chitin microfibrils in cell walls may be arranged in a similar way to that observed for synthetic polymers such as polyurethane which contain rigid segments formed through hydrogen bonding of the urethane groups connected by soft amorphous chains (Krol, 2007). In the case of chitin, the soft chains linking rigid chitin microfibrils could be provided by sufficient lengths of non-microfibrillar chitin, or by β(1,6)-glucan linked to GPI-anchored CWPs and β(1,3)-glucan. Also, it is known that cell wall proteins that are normally attached to β(1,3)-glucan may become attached to the chitin-based endoskeleton when β(1,3)-glucan is damaged (Popolo et al., 2001). Such a cantilevered arrangement would provide mechanical rigidity whilst maintaining the flexibility to rapidly remodel the cell wall when required and would be consistent with proposal that it has viscoelastic properties that is compliant to vesicle transport (Walker et al., 2018) and can act as a sensor that triggers stretch-activated ion channel activity (Brand et al., 2007, Watts et al., 1998). In our model, we have also depicted a chitin synthase protein in the cell membrane. This was based on the structure of a cellulose synthase subunit (Morgan et al., 2013) as the crystal structure of any chitin synthase has not been determined.

### 4.2 β(1,3)-glucan

In the refined model, β(1,3)-glucan is predominantly represented as triple helices with some single helices. The helical conformation of the β(1,3)-glucan molecules is important because it affords the cell wall considerable elasticity (Klis et al., 2009). The size of the helices in our refined model was based on a model of the crystal structure of β(1,3)-glucan (Deslandes et al., 1980, Kobayashi et al., 2013), with six glucose residues per full turn of the helix. Each glucose advances the chain 2.9 Å (0.29 nm), and the diameter of the helix is 14.4 Å (1.44 nm). The organisation of the helices in our refined model was determined by tracing the regular network of interconnected fibrils observed in the inner layer of the cell wall in our 3D cell wall model (e.g. Fig. 1D). Although not shown, the β(1,3)-glucan molecules are branched and will therefore have multiple non-reducing ends which are the attachment sites for the GPI-anchored CWPs via β(1,6)-glucan linkages (Kapteyn et al., 2000). On the basis of their β-glucan masking/shaving work, (Childers et al., 2020) have proposed that some β-glucan chains also extend into the mannan layer.

### 4.3 β(1,6)-glucan

The precise structure of β(1,6)-glucan is difficult to determine because it a small polymer (15–20 monomers) which is highly branched and lacks a regular structure (Klis et al., 2009). In our refined model, we represented the GPI-anchored CWPs as linked to both β(1,3)-glucan and chitin via β(1,6)-glucan linkages (Gonzalez et al., 2009a, Kollár et al., 1997).

### 4.4 Mannosylated cell wall proteins

In our model, mannosylated CWPs are represented throughout the inner cell wall layer with their *N*-linked *manno*-oligosaccharide chains protruding outwards and beyond the inner layer of the cell wall, ultimately forming the outer fibrillar layer of the wall. The presence of sub-cell surface mannoproteins was suggested by our observations (Fig. 2C; Table 3) and was based on the accessibility of CWPs to pronase (Fig. 4). There are two types of CWPs which are attached to the *C. albicans* cell wall, GPI-anchored CWPs and Pir (protein with internal repeats) proteins. Although we did not directly test where specific CWPs are localised in our analysis, immuno-gold labelling of TEM sections of *C. albicans* cells performed by others are in agreement with our results. For example, cells stained with anti-Pir2 antiserum indicated that the Pir proteins are embedded throughout in the inner layer of the yeast cell wall (Kapteyn et al., 2000). There is also supporting evidence that some GPI-anchored proteins (e.g. Ecm331) are retained in the plasma membrane (Klis et al., 2009) which is again consistent with our detection of some staining of the plasma membrane with the anti-mannan antibody (Table 1). Also, in support of this model, immunogold staining of the plasma membrane and inner cell wall layer was observed using an anti-*Candida* monoclonal antibody (mAb AB119) which likely recognises the core *N*-mannan structure (Rudkin et al., 2018). However, (Rudkin et al., 2018) also show an enrichment of staining at the interface of the outer fibrillar and inner cell wall layers for a range of mAbs which recognise proteoglycan epitopes, suggesting that many GPI-anchored CWPs are located in this region.

The GPI-anchored proteins make up around 88% of all CWPs, and of these, 90% are attached to β(1,3)-glucan and 10% to chitin when cells are grown in conditions similar to which we performed our imaging (Kapteyn et al., 2000). A typical GPI-protein is made up of a *C*-terminal stalk region which is *O*-mannosylated and an *N*-terminal globular domain which is *N*-glycosylated (Kapteyn et al., 2000). The CWPs depicted in our refined model are to-scale representations based on crystal structures of the globular domains of GPI-anchored CWPs (e.g. Als3 (chrome yellow), Als2-9 (light olive green), Sod5 (mint green), Sap1 (lemon yellow) and Sap3 (sky blue)), cell wall remodelling proteins (e.g. *endo*-glucanase (beige), *exo*-glucanase (pink) and chitinase (orange)), and a Pir protein (turquoise), to which *N*-mannans are attached. The dimensions of the *N*-mannan fibrils have been drawn based on the analyses of individual mannan fibrils from the 3D cell wall model (Fig. 2E; Fig. 5). There is no crystal structure available for the stalk region of GPI-anchored proteins, and so the length of these has been estimated but takes into account the reported range of atomic force microscopy (AFM) rupture distances of 10 – 200 nm when Conconavalin A-coated tips have been used to pull on mannosylated CWPs in the *C. albicans* cell wall (Beaussart et al., 2012). We present these stalks as being decorated with their stabilising *O*-linked mannans.

### 4.5 Thickness of the cell wall layers

The thickness of the inner and outer fibrillar layer of the cell wall presented in the refined model is based on measurements from the 3D model of the cell wall (Fig. 1C) which is for a *C. albicans* wild type strain growing exponentially in the yeast form in YEPD medium. It is important to remember that the thickness of each cell wall layer varies under different growth conditions (Klis et al., 2014). For example, cells grown on lactate have a thinner cell wall than cells grown on glucose (Ballou et al., 2016, Ene et al., 2012a), and this correlates with large changes to the cell wall proteome (Ene et al., 2012b). The thicknesses of the cell wall layers were also altered in some mutant strains affected in the synthesis of cell wall components, e.g. *och1*Δ (Fig. 3B), but not others, e.g. *chs3*Δ and *pmr1*Δ (Fig. 3D, E). The relationship between the length of the mannan fibrils and the amount of mannan in the cell wall in glycosylation mutants was clear: less mannan correlated with shorter fibrils. This is consistent with analyses of *mnn2* family mutants which have shorter *N*-mannan fibrils (Hall et al., 2013). However, increases or decreases in the amount of chitin and β(1,3)-glucan in the cell wall had no definitive bearing on the thickness of the inner layer of the wall.

We therefore hypothesise that the thickness of the inner layer of the cell wall is not determined solely and directly by the amount of β(1,3)-glucan and chitin. Instead wall thickness may be determined by the CWPs that are present, how they are linked to the β(1,3)-glucan and chitin, and how these linkages are remodelled and space-filling relationships that reflect the way in which the primary components are cantilevered. Supporting this, there is evidence of increased coupling of GPI-anchored CWPs to chitin via β(1,6)-glucan linkages and an increase in Pir proteins in the cell walls of *N*- and *O*-glycosylation mutants (*mnn9* and *pmt1*; Kapteyn et al., 2000). In addition, certain carbohydrate-active CWPs (e.g. transglycosylases) can remodel the cell wall rapidly in response to osmotic stress (Ene et al., 2015). This model of a flexible cantilevered chitin-glucan skeleton suggests further experiments that might predict that the water context and viscoelastic properties of the wall are influenced by cell wall stretch. We know that the wall is compliant to vesicular traffic despite the hydrodynamic radius of the vesicles such has the AmBisome liposome being substantiality larger that than the theoretical cell wall porosity (Walker et al., 2018). The model presented suggest ways in which local compliance in the cantilevered network may allow for such *trans*-cell wall vesicular traffic. These observations portray the cell wall as a viscoelastic structure in which the inner wall defines cell shape and anchors the CWPs predominantly as a basal layer below the *N*-mannan front of fibrils in the outer cell wall.

### 4.6 Conclusion

In summary, our refined model of the *C. albicans* cell wall represents the organisation of the cell surface components of this major fungal pathogen much more precisely than in previous models, and represents the first attempt to generate a genuinely scalar model of any fungal cell wall. Cell surface components encode elements with physiologically critical roles including ligands for immune receptors, adhesins, sites of amyloid formation, hydrophobic clusters and determinants of cell wall porosity, enzymes that modify the properties of the cell wall and are involved in nutrient acquisition, structures that confer protection against endogenous toxins, and enzymes, inhibitors and antifungal agents that act against the cell wall (reviewed in Erwig and Gow, 2016, Foderaro et al., 2017, Garcia-Rubio et al., 2019, Gonzalez et al., 2009a, Gonzalez et al., 2009b, Gow et al., 2017, Hopke et al., 2018). This model will provide a clearer rationale for the design of experiments that investigate these properties, and for those motivated by an understanding of how the fungus interacts with abiotic and cellular substrates. There are still many unanswered questions relating to the structure and biosynthesis of the cell wall that are concerned with the mechanism of cell wall synthesis, assembly and remodelling during growth, and how it adapts to environmental stresses, different niches inside the host, attack from the host immune system, exposure to antifungal agents, and competitive interactions with the bacteria and other organisms in the human microbiota. This new model therefore provides a robust platform to facilitate the testing of hypotheses relating to the structure–function relationships of the cell wall that underpin the pathobiology of this fungus.

## CRediT authorship contribution statement

**Megan D. Lenardon:** Conceptualization, Methodology, Formal analysis, Investigation, Writing - original draft, Writing - review & editing, Visualization, Supervision, Funding acquisition. **Prashant Sood:** Methodology, Formal analysis, Investigation, Writing - review & editing, Visualization. **Helge C. Dorfmueller:** Investigation, Visualization, Writing - review & editing. **Alistair J.P. Brown:** Writing - review & editing, Supervision. **Neil A.R. Gow:** Conceptualization, Writing - review & editing, Supervision, Funding acquisition.

## Declaration of Competing Interest

The authors declare that they have no known competing financial interests or personal relationships that could have appeared to influence the work reported in this paper.
