# Supplementary material for: Scalar nanostructure of the Candida albicans cell wall; a molecular, cellular and ultrastructural analysis and interpretation
Source: Cell Surf. 2020 Nov 8;6:100047. doi: 10.1016/j.tcsw.2020.100047 (PMC7691183; doi:10.1016/j.tcsw.2020.100047)
Supplement: Supplementary figures 1, 2 & 3 [file mmc1.pdf]

Supplementary Figures 1, 2 and 3: Lenardon et al.

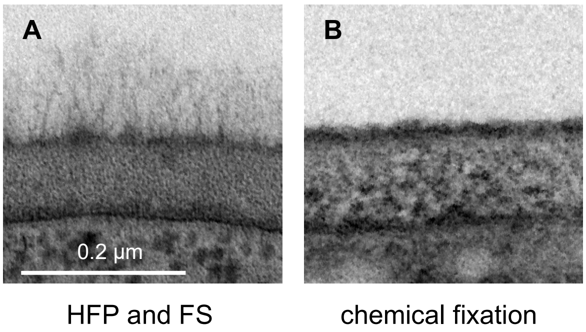

Figure S1: HPF/FS preserves the cell wall ultrastructure better than chemical fixation. A. Cells of a wild type strain (UC820) were grown in YEPD+uri to mid-log phase (6 h) at 30°C, harvested by centrifugation, HPF/FS and embedded in Spurr's resin. **B.** Cells of the wild type strain (CAI-4+CIp10) were grown in YEPD+uri to mid-log phase (6 h) at 30°C, harvested by centrifugation, fixed in 2.5% (v/v) gluteraldehyde in cacodylate for 24 h at 4°C and embedded in Spurr's resin. In both cases, 90 nm sections were stained with uranyl acetate and lead citrate before imaging in the TEM. The scale bar represents 0.2 µm and is the same for both images.

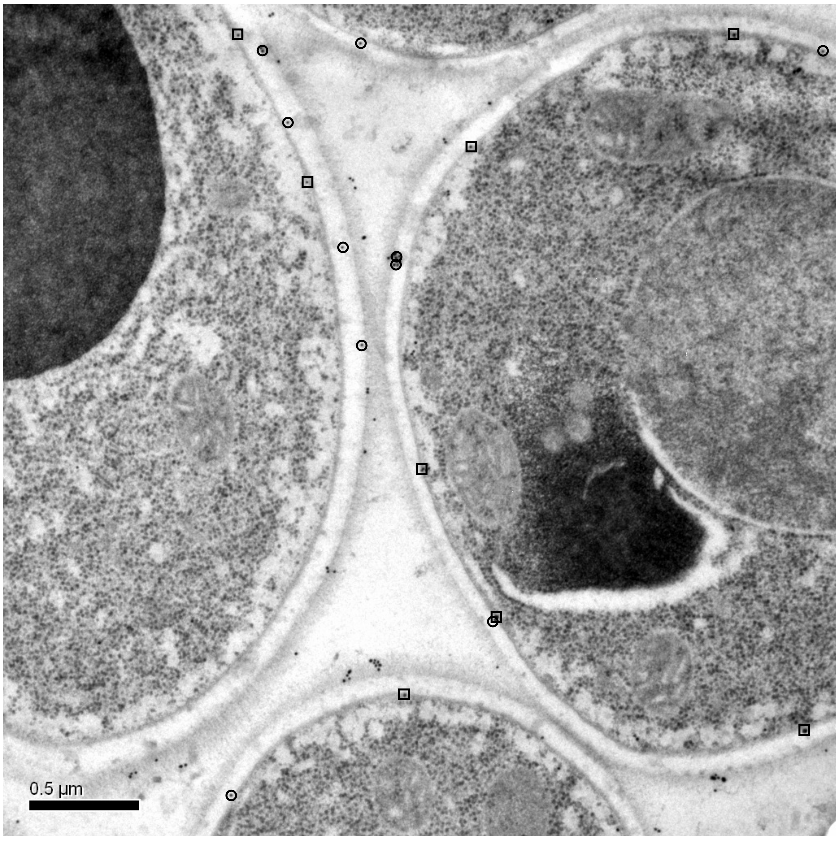

Figure S2: The distribution of mannan in the *C. albicans* cell wall. Cells of a wild type strain (CAI-4 +CIp10) were grown in YEPD+uri to mid-log phase (6 h) at 30°C, harvested by centrifugation, HPF/FS and embedded in HM20 resin. Ultrathin sections (100 nm) sections on copper grids were stained with the anti-mannan scAb, followed by an anti-HuCκ antibody (raised in mouse) and a gold-labelled anti-mouse IgG (40 nm gold particle). Grids were dried and then stained with uranyl acetate and lead citrate before imaging in the TEM. Gold particles staining the inner layer are marked with circles and gold particles staining the cell membrane are marked with squares. The scale bar represents 0.5 µm. In this individual image, 80 (total) gold particles stained the cell wall, of which 56 (70%) bound the outer fibrillar layer, 17 (21%) bound the inner cell wall and 7 (9%) bound the cell membrane. The number of cell walls counted in this image was scored as 4, giving an average of 20 gold particles per cell wall.

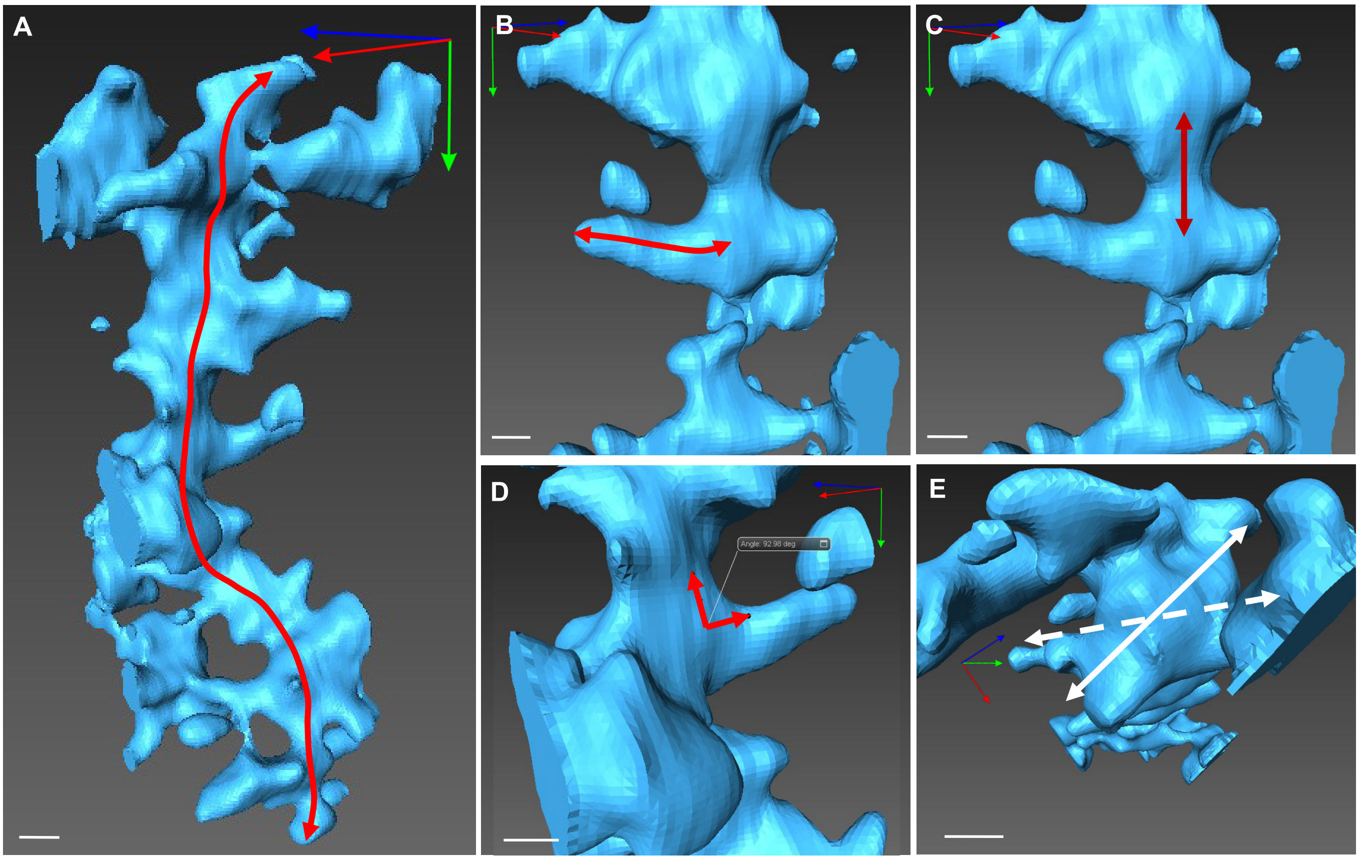

Figure S3: Models and measurements of individual mannan fibrils. A. An example of an intact and discrete mannan fibril which was dissected out, segmented, reconstructed and modelled. Total fibril length was measured from the top to the base of the fibril along its spine in 3D voxel space (represented approximately by the red arrow). **B.** Branch length was measured from the tip of each lateral branch to the centre of the spine (red arrow). **C.** Inter-branch length was measured as the distance between adjacent branches emerging from the same spine (red arrow). **D.** The branch angle was measured as the angle at which each branch emerges from the spine when visualising the fibril front on. **E.** The branch arrangement refers to the angle at which the branches emerge from the spine when looking down the shaft of the fibril (white arrow: coronal plane; dashed arrow: angulated plane in which branches were aligned). In all panels, scale bars represent 2 nm, and the x, y and z axes are shown in red, green and blue.
