# Supplementary material for: Scalar nanostructure of the Candida albicans cell wall; a molecular, cellular and ultrastructural analysis and interpretation
Source: Cell Surf. 2020 Nov 8;6:100047. doi: 10.1016/j.tcsw.2020.100047 (PMC7691183; doi:10.1016/j.tcsw.2020.100047)
Supplement: Supplementary figure 4 [file mmc2.pptx]

## Slide 1
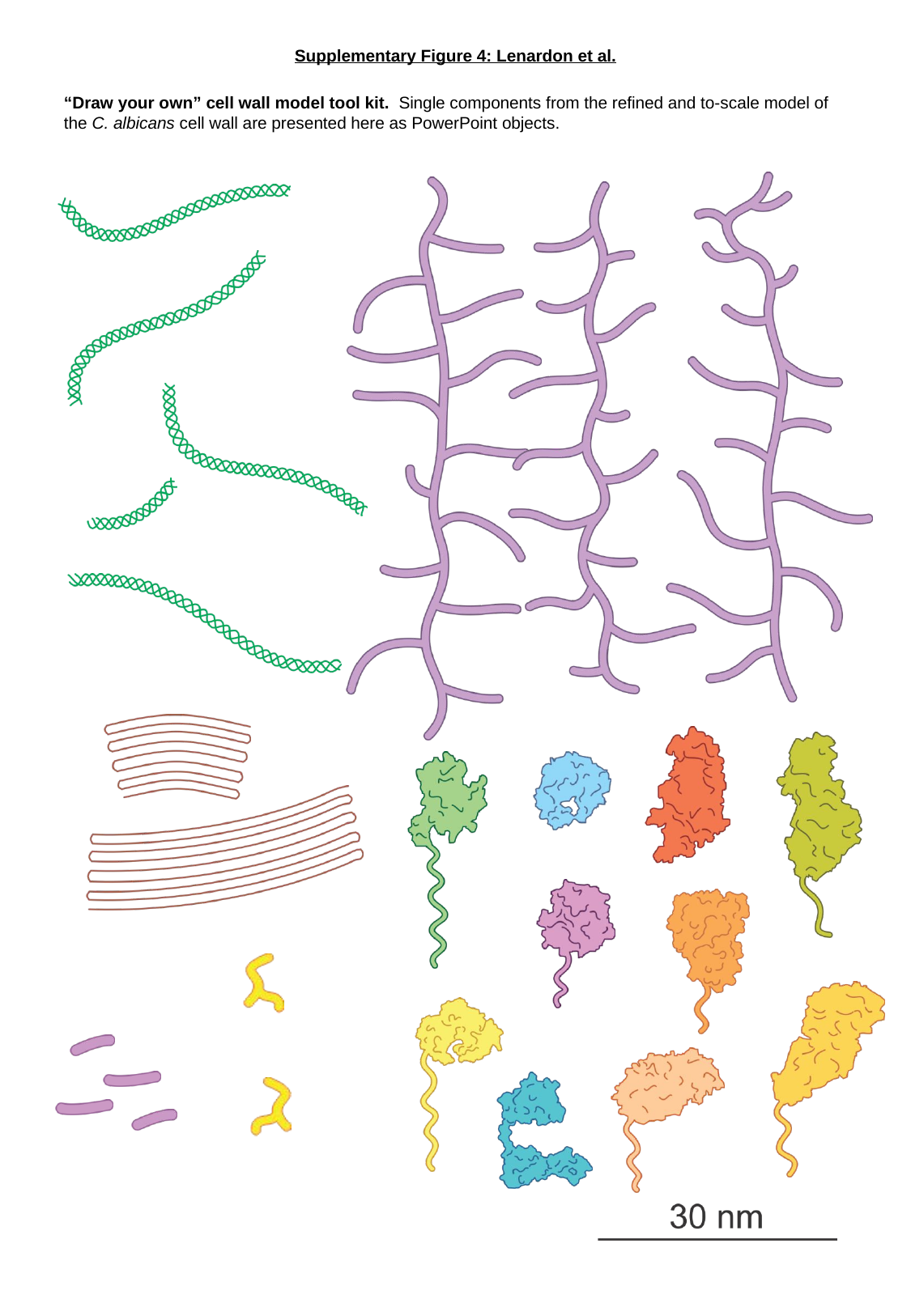

Supplementary Figure 4: Lenardon et al.
“Draw your own” cell wall model tool kit. Single components from the refined and to-scale model of the C. albicans cell wall are presented here as PowerPoint objects.
